# Study protocol of brief intervention using gene polymorphism information for excessive drinking among Japanese college students and adults aged 20–30 years: a randomized controlled trial

**DOI:** 10.1186/s13063-022-06645-7

**Published:** 2022-08-26

**Authors:** Yukiko Owaki, Hisashi Yoshimoto, Go Saito, Takahiro Goto, Satoshi Kushio, Akihiro Nakamura, Yusuke Togo, Kazumasa Mori, Hideki Hokazono

**Affiliations:** 1 Department of Primary Care and Medical Education, Faculty of Medicine, University of Tsukuba, Tsukuba, Japan; 2 Department of Community General Medicine, Faculty of Medicine, University of Tsukuba, Tsukuba, Japan; 3 General Medicine and Primary Care, University of Tsukuba Hospital, Tsukuba, Japan; 4 Graduate School of Comprehensive Human Sciences, Doctoral Program in Medical Sciences, Department of Disease Control Medicine, University of Tsukuba, Tsukuba, Japan; 5 Sanwa Laboratory, Sanwa Shurui Company, Usa, Japan

**Keywords:** Excessive drinking, *ALDH2*, *ADH1B*, Gene polymorphisms, Japanese, College students, Adults aged 20–30 years, Randomized controlled trial

## Abstract

**Background:**

The alcohol-metabolizing enzyme aldehyde dehydrogenase 2 (*ALDH2*) is a carcinogenic acetaldehyde-degrading enzyme, and its low activity is a genetic constitution peculiar to East Asians. People with low alcohol dehydrogenase 1B activity (ADH1B*1/*1 genotype) have a high risk of developing head and neck cancer and alcoholism. The study aims to evaluate the effectiveness of brief interventions for excessive drinking among college students and adults in their 20s, including information on five constitutions that combine the *ALDH2* and *ADH1B* genotypes.

**Methods:**

Participants comprised university students and staff aged 20–30 years who had consumed ≥40 g (males) or ≥20 g (females) of pure alcohol; they were classified into intervention and control groups using a simple randomization method. Participants anonymously filled out questionnaires linked to identification numbers and recorded the drinking days and amounts on the drinking calendar. The intervention group will then be tested for genotype testing using saliva (5 types of combinations of *ALDH2* and *ADH1B* enzyme activities); the result report will arrive approximately 1 month later. We will conduct a 30-min face-to-face or online intervention. The control group will be merely given the conventional materials, and genetic testing will be performed voluntarily after 6 months (end of study). The intervention group will undergo questionnaire surveys 1 month after the intervention and 3 and 6 months after baseline. Questionnaire surveys will be conducted 1, 3, and 6 months after baseline for the control group. The average amount of drinking before and after the intervention, attribute/baseline data between the two groups, and time-series data were compared using various analysis tools. For interventions, we engaged in dialog based on intervention materials that added genotyping content to the existing materials, result reports, baseline data, and drinking calendar records. Participants’ ingenuity is respected to support their drinking behavior and goal setting.

**Discussion:**

Individual information on the genetic makeup of alcohol-metabolizing enzymes provided during the intervention is more personal and objective than general health information, especially in Japan, where the *ALDH2* low activity rate is high. This information may be useful for health care and precautionary measures.

**Trial registration:**

R000050379, UMIN000044148, Registered on June 1, 2021.

Scientific Title: Examination of simple intervention using genetic polymorphism information for excessive drinking.

## Background

A genetic constitution with a low *aldehyde dehydrogenase 2* (*ALDH2*) activity is atypical in East Asians [1]. In Japan, less than 10% of the population is *ALDH2* homodeficient, whereas 30–40% is *ALDH2* heterodeficient [2]. In Japan, Taiwan, and China, *ALDH2* heterodeficient drinkers have a particularly high risk for esophageal and head and neck cancer, and *ALDH2* heterozygous deficiency is strongly associated with multiple carcinogenesis and juvenile carcinogenesis [2]. The results of all Japanese and Chinese case–control studies on the association between esophageal cancer and *ALDH2* heterozygotes deficiency showed that *ALDH2* heterozygous deficiency increased the risk of esophageal cancer in drinkers (5.3–16.4 times the adjusted odds ratio). This tendency is not limited to heavy drinkers, and recent studies have shown a similar tendency in women [3]. ALDH2 * 2 allelic carriers with low *ALDH2* activity were reported to have more than 10-fold higher blood acetaldehyde levels after alcohol intake than wild-type homozygotes (* 1 / * 1) [4].

In the USA, alcohol consumption among young adults aged 18–44 years is a risk factor for cancer development, indicating that alcohol screening and intervention in this age group are crucial [5]. Additionally, the relationship between gene polymorphisms in alcohol-metabolizing enzymes and the risk of alcohol-related health problems includes the risk of developing acute alcohol intoxication with inactive *ALDH2*. *Alcohol dehydrogenase 1B* carriers (ADH1B * 1 / * 1 genotype) have been reported to be at increased risk of alcohol dependence [2], as well as an increased risk of head and neck cancer due to excessive alcohol consumption [6–8]. Specifically, *ADH* gene polymorphisms also affect alcohol consumption, with ADH1B * 1 / * 1 having slower ethanol metabolism than * 2 allelic carriers and long-lasting drunkenness [4]. The combination of ADH1B * 1 / * 1 and ALDH2 * 1 / * 1, which is common in Caucasians, tends to increase alcohol consumption. Higuchi et al. reported that the risk of alcoholism was approximately 6–10 times higher [9]. However, the combination of ADH1B * 1 / * 1 and ALDH2 * 1 / * 2 also has a high risk of alcoholism. Of the 26% people who drank ≥60 g of pure alcohol per day [10], 13% had alcoholism [9] and ALDH2 * 1 / * 2. Looking at ADH1B * 1 / * 1 alone, the risk of esophageal cancer in drinkers was 2.71–3.22 times higher; however, the combination of ADH1B * 1 / * 1 and ALDH2 * 1 / * 2 had 12.45 times higher risk [11]. In any case, excessive drinking is harmful to the physical and mental health. In particular, ALDH2 * 2 allele carriers are considered more harmful due to the accumulation of acetaldehyde [4]. Therefore, it is important to understand the genotypes of alcohol-metabolizing enzymes and make brief interventions for each genotype in terms of preventing cancer, addiction, and acute alcohol intoxication.

On the contrary, a systematic meta-analysis review in 2016 examined the effect of communicating genetic risk of disease on the risk-reducing health behaviors based on the results of multiple literatures [12]. The overall conclusion of the meta-analytic review stated that the expectation that transmission of DNA-based risk estimates would alter behavior was not supported by existing evidence [12]. In this review, the use of alcohol was examined based on three studies [13–15]. Those three studies assessed self-reported alcohol use, with genetic risks communicated for cancers [13, 15] and cardiovascular diseases [14]. Comparisons were between DNA-based risk estimates versus no risk estimates. Pooled data (*n* = 239) revealed no evidence of an effect of DNA-based risk communication on reducing alcohol use [12].

However, each of those studies examined alcohol use, and a 2010 American evaluation study [13] used the Internet to examine the genotype and health risk feedback effects on *ALDH2*. Results showed that the frequency and amount of drinking after 30 days were significantly reduced in participants with low *ALDH2* activity who received genetic feedback, which they highly appreciated [13]. This study population was composed of 200 college students (males, 46.5%; mean age, 20.2 years [SD = 1.5]), and they were assigned randomly to a group with genetic feedback (*n* = 100) or a group with attention without genetic information. The *ALDH2* genotype was analyzed using blood samples [13]. In addition, in a 2006 report [15], 329 male employees who wanted to know the results of the ALDH2 genotype were randomly divided into two groups. One is the “notified group” (*n* = 157), and the other is the “unnotified group” (*n* = 172). Patients in the “notification group” were notified of the results of the *ALDH2* genotype diagnosis, and drinking habit and liver function test data were obtained before and after notification of the *ALDH2* genotype. As a result, no significant change was found in the drinking frequency, and liver function test data did not decrease in any group before and after genotype notification. However, in patients with *ALDH2* activity genotype ALDH2 * 1 / * 1, weekly drinking tended to increase than that before notification, and *ALDH2* low-activity genotype ALDH2 * 1 / * 2 patients tended to increase in the unnotified group, but decreased in the notified group. No significant differences were noted in the results, but further studies and increased sample size suggested that genetic testing might be useful [15]. In addition, Hanna-Leena et al. [14] reported a long-term follow-up study in 2018 on the disclosure of personal genetic risk information (APOE: apolipoprotein E) for cardiovascular diseases to promote a healthy lifestyle in Finland [16]. In conclusion, receiving information on increased personal genetic risk (carrier status of APOE ε4) for cardiovascular diseases provided the motivation for improvements in health behavior. The resulting changes, while modest, in most cases remained visible even after a few years [16].

A recent Korean research [17] introduces a previous study by Japan [18]; after drinking the same amount of alcohol, ALDH2 * 2 homozygotes and heterozygotes showed 18- and 5-fold higher blood alcohol levels than ALDH2 * 1 homozygotes, respectively, suggesting that interventions in alcohol use should be specified individually for each genotype. The paper emphasizes the role of *ALDH2* polymorphisms in alcohol-related health problems, requires consideration of *ALDH2* genotypes in alcohol prevention programs, incorporates genetic information, and informs people about alcohol consumption. They suggested an urgent need for a strategy that would provide the basis for evidence to support evidence-based decision making [17].

In 2009, Ishikawa et al. reported the intervention effect of correcting the drinking behavior problem in Japan by using the gene polymorphism information of alcohol metabolism [19], and they provided health guidance by adding the gene polymorphism information of *ALDH2*. They discovered that the average daily drinking after 6 months was significantly lower in the intervention group than in the control group without genetic information and that the ratio of drinking behavior patterns classified by drinking amount and frequency was significantly improved [19]. They also classified *ALDH2* into three types: (1) normal type homo (ALDH2 * 1 /ALDH2 * 1 type); (2) normal type and mutant type hetero (*ALDH2*); and (3) * 1 /ALDH2 * 2 type, variant homo (ALDH2 * 2 /ALDH2 * 2 type). The first two were used for male adults in Japanese offices during blood sampling tests. Ishikawa, who is also an industrial physician at the business establishment, provided individual health guidance [19]. The intervention effect was based on the previous questionnaire on drinking habits and was compared with the results on drinking habits using the same questionnaire 6 months later. In 2017, Taketani et al. investigated 32 university students and graduate students aged 20 to 29 at one university as young adults in Japan. They examined the difference in alcohol education usefulness between the group that was notified of the genotype of enzymes (*ADH1B*, *ALDH2*) and the group that was not notified (hereinafter referred to as the “non-notification group”) by comparing the results of the questionnaire designed for their study [20]. They found that the amount of change in the knowledge of alcohol-related problems after alcohol abuse education was not significantly different between the two groups. However, presenting a scientific constitution using genotypes may improve their drinking behavior, including reconsidering their future drinking behavior [20].

One of the effective intervention methods presented by the National Institute on Alcohol Abuse and Alcoholism for overdrinking among college students is a simple method known as Brief Alcohol Screening and Intervention for College Students (BASICS). BASICS includes initial screening to identify high-risk drinkers, subsequent baseline assessments to generate individualized feedback, and one-on-one meetings with trained facilitators to confirm feedback [21]. According to a 2020 report of a randomized clinical trial conducted in India by Kumar et al. [22], out of 793 students from two universities screened using the Alcohol Use Disorder Identification Test (AUDIT), 130 had an AUDIT score of 8–19. These 130 participants were randomly assigned to two groups: an experimental group (*n* = 64) with individually tailored brief intervention by trained nurses and a control group (*n* = 66), which only received general advice. Intervention effectiveness was assessed 3 months after one session of screening and brief intervention (SBI) and compared in terms of the changes in the AUDIT score and the percentage of students transitioning from the high-risk zone to the low-risk zone between the two groups. According to the results, SBI provided by individual-based nurses reduced the severity of problems or hazardous drinking in the short term (after 3 months of intervention), but the estimated effect size was small (0.16) [22]. In addition, face-to-face individual interventions are costly and difficult to disseminate on a large scale; thus, the effectiveness of individual short-term alcohol interventions provided by computers for heavy-drinking college students and young adults has also been tested. In a 2015 US review, the effects of computer delivery interventions were positive but small [23].

Integrating the results of previous studies above, interventions for excessive drinking in college students and young adults will be more effective and enduring with individual brief interventions using gene polymorphism information for alcohol metabolism than previous brief interventions. Moreover, this intervention may enhance the effect of correcting excessive drinking, especially in Japan, where *ALDH2* is often inactive. This randomized controlled trial aims to clarify that.

This study examines individual brief intervention methods, including information about *ALDH2* and *ADH1B* genotypes (5 types) for excessive drinking among university students and staff aged 20–30 years as young adults. The study is currently recruiting participants, collecting data, and conducting some analysis. To verify the effectiveness of this intervention, this paper reports the study protocol.

## Methods

### Overview of the study design

The study population is divided into two groups: the intervention group (which performs individual interventions for drinking habits, including the results and points to note concerning genotypes determined using saliva) and the control group (which has no constitutional examination during the study period, providing only conventional teaching materials). This study is an open-label, randomized controlled trial. We chose an open-label trial because participants can be distinguished between the intervention group, who undergoes the test of genotypes at baseline, and the control group, who wishes to test for genotype after 6 months of study. In principle, the outcome evaluator does not provide interventional care, and only the outcome evaluator is blinded.

**Fig. 1:**
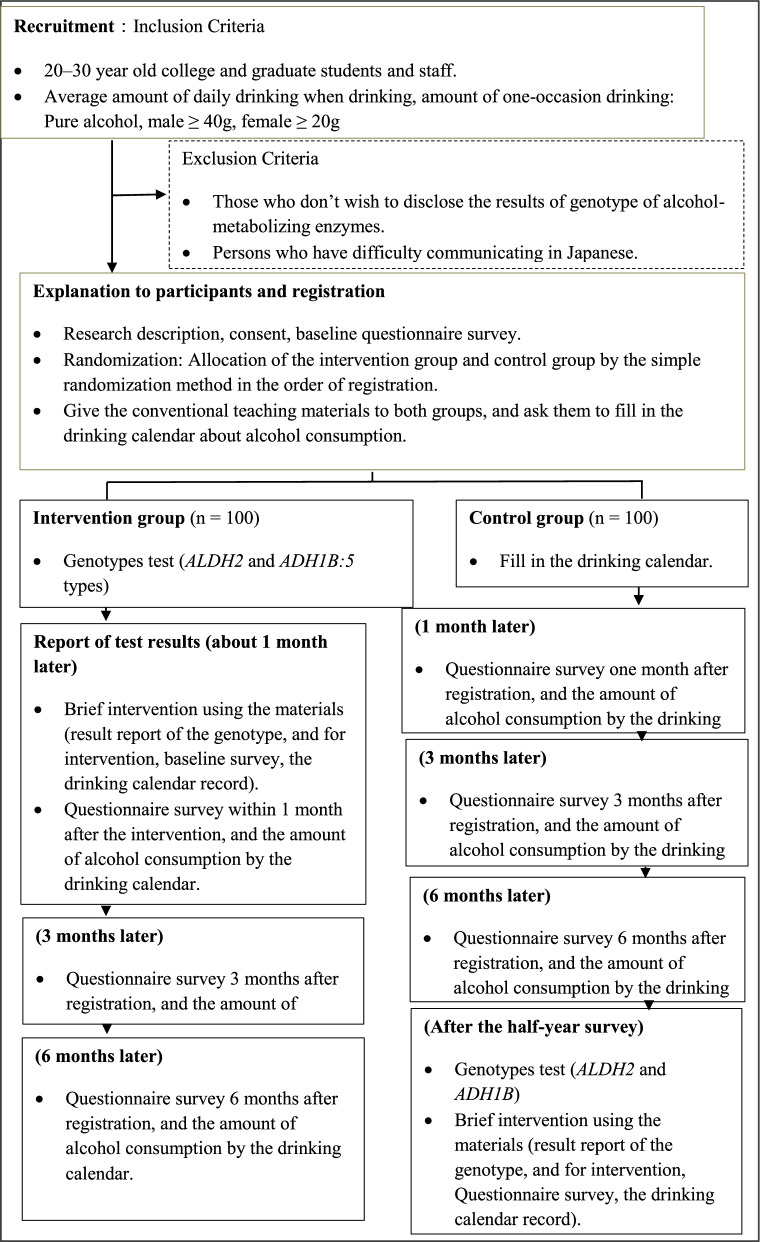
Study flow chart. *ALDH2* aldehyde dehydrogenase 2, *ADH1B* alcohol dehydrogenase 1B

Figure 1 shows the study flow chart, and Table 1 lists the components of the five genotypes of alcohol-metabolizing enzymes and the major health risks.

**Table 1:** Composition of five genotypes of alcohol-metabolizing enzymes and the health risks (supervised by National Hospital Organization Kurihama Medical Center. Partial excerpt) [1]

Aldehyde dehydrogenase	Alcohol dehydrogenase	Type classification of this research	Comments on constitution and health risks
ALDH2 *1/ *1 (activity)	ADH1B *1/ *1 (low activity)	A (3%)	The highest risk of alcohol addiction
	ADH1B *1/ *2 (activity)	B (50%)	Decomposition of alcohol and acetaldehyde is fast. Be careful not to drink too much for your health.
	ADH1B *2/ *2 (high activity)		
ALDH2 *1/ *2 (low activity)	ADH1B *1/ *1	C (3%)	The decomposition of alcohol and acetaldehyde is slow, and the cancer risk is high, owing to acetaldehyde.
	ADH1B *1/ *2	D (40%)	Face turns reddish immediately. Nausea and other discomfort can occur. Symptoms and health problems are likely to occur.
	ADH1B *2/ *2		
ALDH2 *2/ *2 (Inactive)	ADH1B *1/ *1	E (4%)	Drinking is intolerable because aldehyde cannot be decomposed. Very small amounts of alcohol are prone to unpleasant symptoms such as hot flushes, drowsiness, palpitation, and nausea. Even a small amount of alcohol is at risk of acute alcohol intoxication.
	ADH1B *1/*2		
	ADH1B *2/ *2		

### Study setting

This study is being conducted at a national university in the metropolitan area in Japan and is recruiting participants. The university is a comprehensive university, including medical departments. In 2021, the total number of students and faculty members were 16,542 and 4,608, respectively.

### Participants

We included students and graduate students, and staff aged 20–30 years at the University of Tsukuba. The average amount of daily or one-occasional drinking is more than 40 g of pure alcohol for males and more than 20 g for females. They provided their consent to participate in this research.

The exclusion criteria are those who did not wish to disclose the results of the alcohol constitution test and those who have difficulty communicating in Japanese. Participation is voluntary, and participants will be withdrawn if they request to stop participating.

### Sample size

With reference to previous studies [13, 22], the total sample size was 199, calculated with a power size of 0.2, an *α* error of 0.05, and a power of 0.8 using the power analysis software (G * Power3) [24]. In total, we include 200 participants (100 in the intervention group and 100 in the control group).

### Recruitment

With permission, recruitment posters are displayed on the main bulletin boards of the university by researchers, mainly the first authors. The procedure of accessing the written explanation and consent form is given to the target person who is contacted by a researcher (mainly the first author) via e-mail. Through snowball sampling, we also ask an acquaintance of the participants to introduce the recruitment poster. We aim to reach the number of targets within 1 year from the start of recruitment, but if there is a shortage, we will continue to make posters at the university and increase the number of recruitment by snowball sampling.

### Screening and informed consent

Simultaneously, as the consent procedure, a self-administered questionnaire survey of baseline data are conducted, and the explainer confirmed the drinking status and average drinking amount during questionnaire collection. The procedure for explanation and consent is mainly conducted by the first author. With written consent, the intervention group is tested for alcohol-metabolizing enzyme genotype using saliva. The explainer collect and mail the sample to the testing institution on the day of the test. On the consent form, participants will be asked if they agree to the use of their data, should they choose to withdraw from the trial. Participants will also be asked for permission by the research team to share relevant data with people from the universities participating in the study or, if necessary, from regulatory agencies. If the consent and permission are confirmed, the data can be used and shared. This study does not include collecting biological specimens for storage.

During the time of study planning and implementation, measures against coronavirus disease 2019 (COVID-19) are required. Informed consent is supposedly obtained in person; however, depending on the participant’s wishes and place of residence (during homecoming, etc.), explanation, consent, and inspection procedures are performed online and by mail, as necessary.

### Randomization

In randomization, we simply used a computer-generated random number table, collate participants with anonymizing identification numbers, and assign them to the intervention group or the control group (100 participants per group). The first author mainly marked the random number table as the identification number of the intervention group before the start of recruitment and assigned it in the order of registration depending on the presence or absence of the mark.

### Measures

From the self-written questionnaire anonymized by an identification number, basic attributes (age, sex, main areas of learning, living with or without family, work situation, participation in circles, and hobby activities) and evaluation items will be investigated. For the intervention group, we will collect data 1 month after the intervention, and 3 and 6 months after the questionnaire survey at the time of consent procedure.

At the same time as the questionnaire survey, the content and amount of drinking will be recorded in a drinking calendar for half a year anonymized by the identification number. For the questionnaire and drinking calendar, the researchers (mainly the first author) will ask the participants to answer by e-mail, and the data from the returned attached file will be collected. We will ask for a response within a week from the survey point, and if there is no response, we will optionally request a response by reminder email within a month.

### Primary outcome measures

According to participants’ average amount of daily drinking (g = pure alcohol, 1 drink = 10 g of pure alcohol), we will compare the effects of interventions by adding information on the genotypes of alcohol-metabolizing enzymes to the content of conventional teaching materials.

The questionnaire provides the following examples of the drink conversion table for alcoholic beverages (Sake: 15% alcohol concentration, 180 ml = 2 drinks; beer: 5% alcohol concentration, 500 ml [medium bottle/medium mug or can beer] = 2 drinks; whiskey brandy: 43% alcohol concentration, 60 ml [double] = 2 drinks; shochu: 25% alcohol concentration, 100 ml [cup half ] = 2 drinks; canned shochu: 7% alcohol concentration, 350 ml = 2 drinks; cocktails, fruity taste, etc.: 5% alcohol concentration, 350 ml [canned] = 1.5 drinks, 500 ml [canned] = 2 drinks; wine: 12% alcohol concentration, 150 ml (glass ) = 1.5 drinks; plum wine: 12% alcohol concentration, 90 ml [small glass] = 1 drink). We ask the answer of the average number of drinks on the drinking day of the past month.

A similar example is shown in the drinking calendar, and the drinking contents (alcohol type and alcohol concentration) and amount on the day of drinking will be recorded for half a year. From the record of the calendar, the sum of the drinking amount (pure alcohol amount) and the number of drinks is calculated using the following generally accepted formula: amount of liquor (ml) × degrees or %/100 × 0.8 (specific gravity) = pure alcohol content (g).

### Secondary outcome measures

As the secondary outcomes, screening test (AUDIT-C and AUDIT) scores, awareness of participant’s flushing reaction (simple flushing questionnaire method two items) [3], recognition of constitution strength against alcohol, and recognition of drinking amount by 5-step Likert scale are examined. AUDIT, which consists of ten self-written questions, was prepared by the World Health Organization as a screening method for excessive drinking. Hiro et al. verified the effectiveness and validity of the Japanese version of the questionnaire [25], and in the current guidance for AUDIT SBI in Japanese health guidance, an AUDIT score of 7 points or less is considered low-risk drinking, whereas an AUDIT score of 8 points or more suggests excessive drinking [26]. AUDIT-C is a simple screening test (maximum score of 12 points) composed of the first three items of AUDIT, and in Japan, 6 points or more for males and 4 points or more for females are considered excessive drinking, corresponding to 8 points or more of AUDIT [27].

Furthermore, we will investigate the five stages of change in drinking behavior: precontemplation, contemplation, preparation, action, and maintenance [28]. Self-recognition of the genetic constitution of alcohol-metabolizing enzymes will also be conducted. In addition, we will evaluate the modified Perceived Health Competence Scale (modified PHCS) Japanese version [29], the impact on learning by drinking over the past month, sleep condition, the Subjective Fatigue Scale for Young adults (SFS- Y) [30], and recognition of the usefulness of genotype test on alcohol-metabolizing enzymes in health care.

Japan’s Ministry of Health, Labor, and Welfare has listed support technology in the “Standard Health Checkup and Health Guidance Program Definitive Edition” and calls for “indifference, interest, preparatory period, execution period, and maintenance period” as “the preparation stage for behavior change,” and “to support the stage to improve by changing the support method for each stage” (definitive edition p86, revised version p121, 2018 edition p3-2) [31]. The five stages are as follows: (1) indifference period, lack of intention to take action toward behavior change within 6 months; (2) period of interest, willingness to take action toward behavior change within 6 months; (3) preparation period, willingness to take action toward behavior change within 1 month; (4) implementation period, observance of a clear behavior change at less than 6 months only; (5) maintenance period, clear behavior change lasting for more than 6 months [28]. In this study, the survey was conducted at baseline and four points up to half a year.

The modified PHCS Japanese version [29], which has eight items (5-step Lickert scale), was created by Smith, Wallston, and Smith (1995) according to self-efficacy theory as a performance index involved in changes in health-related habits and behavior. Its reliability and validity were verified by Togari et al. [29]. In addition, while alcohol has a hypnotic effect, drinkers are easily awakened because of the decrease in the blood concentration of alcohol and diuretic action after falling asleep; in fact, the association between binge drinking and sleep problems was suggested in young adults in particular [32]. Therefore, the presence or absence of binge drinking (within 2 h, >5 drinks for males and >4 drinks for females) and sleep status (recognition of sleep time and sleep quality: 4-step of Likert scale, difficulty falling asleep, and wakefulness during sleep) in the past month were added to our questionnaire. In addition, fatigue conditions will be investigated using SFS-Y [21], considering that the burden on the liver by excessive drinking leads to fatigue. We will also investigate the effect on class attendance and assignment submission on a 5-step Likert scale. SFS-Y has 24 items (6 subscales: difficulty in intensive thinking, fatigue, decreased motivation, decreased vitality, drowsiness, body discomfort), and it has been proven to be reliable and valid [21].

### Statistical analysis

Baseline data and participant attributes, average drinking amount on drinking days, AUDIT-C and AUDIT scores, and response at each scale of secondary outcome measures at each measurement point will be analyzed by a descriptive statistic.

Furthermore, baseline data, attributes, and changes in the average daily drinking amount (1 drink = 10 g of pure alcohol) before and after intervention are compared with the differences between the two groups. We will also compare the measurement points within the groups and compare the groups in terms of genotypes. For these analyses, we use the *t*-test, paired *t*-test, Wilcoxon rank-sum, chi-square, Mann–Whitney *U*, and Kruskal–Wallis tests as appropriate. The selection of the analysis method depends on the parametric and nonparametric data obtained from the results of descriptive statistics and normality tests to determine. In addition, the change in the average amount of drinking (1 drink = pure alcohol 10 g) in each group is examined by calculating the effect size. Data will be analyzed using IBM SPSS Statistics for Windows version 28. If the intervention is not possible in the intervention group, or if no response will be obtained after 1 month in the control group, it will be excluded from the analysis.

Regarding qualitative data (positive aspects about the alcohol constitution test, reasons, etc.), we will examine them by using an inductive analysis method with qualitative coding. The code extracted to ensure reliability will be analyzed by multiple researchers, including those with qualitative research experience.

## Alcohol-metabolizing enzyme genotype testing and results report

Briefly, the intervention group will read the instructions for the alcohol-metabolizing enzyme test, sign the consent form, undergo saliva collection using a sponge swab from the test kit, and receive a report of the results approximately after 1 month. The inspection contractor will mail the result report to the participants. If face-to-face explanation and inspection are difficult to conduct, the test kit will be mailed to the participants.

In the control group, testing will be conducted on those who wish to be tested after 6 months from baseline. After receiving the test results, their requests will be confirmed, and the intervention will be performed.

Regarding the report of the results of the alcohol-metabolizing enzyme test, we have created a pamphlet that includes contents common to each of the five types of constitutions and the contents for each constitution. The main contents are as follows:

- Participants’ alcohol constitution (what type of constitution from A to E, characteristics by constitution type)
- Mechanism of alcohol decomposition, function of metabolic enzymes, and constitution by combination
- Risks of diseases associated with drinking by alcohol constitution (every five types) and points to remember (comments by the constitution)
- How to interact with and drink alcohol that suits participants’ alcohol constitution, and how to absorb alcohol ingenuity in nutrition and eating and drinking to help slow down the metabolism
- Drinking habit screening test (AUDIT-C)
- Appropriate drinking and liver rest days, proper drinking method, alcohol calorie, and metabolism, obesity, etc.
- Inappropriate drinking: drinking while pregnant/lactating, drunk driving, alcohol harassment, and habitual use/heavy drinking

## Intervention training and intervention

### Intervention training

The intervening researcher is a nurse who had experienced caring for patients with health problems resulting from excessive drinking and who trained in intervention by physicians providing treatment to reduce drinking at the university. Specifically, this nurse will conduct simulated interventions with physicians online and face-to-face, receive advice (approx. 2 h), and observe physician’s interventions in outpatients (approx. 20 people).

### Actual intervention

Participants in both the intervention and control groups can lead a normal life, with no particular restrictions or changes in daily care, which will continue in both study groups.

Interventions will be conducted according to the results of individual test results, baseline questionnaire data, drinking calendar records, and health guidance materials, including conventional teaching materials [33]. The main contents of the health guidance materials are as follows:

- Alcohol action (disinhibition/relaxation, sedation/anesthesia, blood alcohol concentration, and drunken stages and symptoms)
- Mechanism of comfort and discomfort associated with drinking (relationship with neurotransmitters in the brain reward system, action of alcohol-metabolizing enzymes, harmfulness of acetaldehyde)
- Alcohol constitution (what type of constitution from A to E, characteristics, and comments by constitution type)
- Drinking habit screening test (AUDIT-C: ≥6 points for males, ≥4 points for females) and feedback that continuing the current drinking habits may eventually affect the mind and body
- Drinking amount ranking based on the current drinking amount (national standard table of drinking for general adult males and females)
- Generally appropriate amount of alcohol: two drinks for males (20 g of pure alcohol) or less on average per day, one drink (10 g of pure alcohol) or less for females
- The number of people who are vulnerable to alcohol, who are older, and who have a red face when drinking alcohol is even less
- Generally inappropriate drinking (drinking while pregnant/lactating, driving a car (including a bicycle), minor, illnesses, or medications affected by drinking)
- Drinking with a high risk for lifestyle-related diseases: four drinks for males on average per day (40 g of pure alcohol) or above, two drinks for females (20 g of pure alcohol) or above
- Binge drinking: within 2 h, five drinks for males (50 g of pure alcohol) or more, four drinks for females (40 g of pure alcohol) or more
- Information from recent studies: zero daily drinking may be desirable
- Participants’ intentions regarding drinking behavior and drinking habits (continuing or reviewing the current drinking habits is better)
- Participants’ opinion about the advantages and disadvantages of drinking alcohol
- Review on how to drink, the possible goal, and the concrete method
- Examples of self-monitoring: proposal for continuing the drinking diary and drinking calendar recording

During the intervention, the intervening researcher confirms the participants’ perceptions of drinking behavior and drinking amount, their intention to review their habits, and the content they intend to change, and respects the participants’ ingenuity in drinking methods and goal setting.

The intervention time is approximately 30 min as a guide, but it can be extended to 1 h to answer questions, share information with each other, and communicate. At the end of the intervention, the participants are told that they can contact the intervening researcher via e-mail for any questions or consultations, and they can respond to via e-mail as well, as needed. If an interview is requested after the intervention, we will set up an interview at the end of the half-year survey and only follow them up by e-mail. Given that measures against COVID-19 are required during the planning and implementation of this study, the application Zoom was used as the intervention method in accordance with the participants’ wishes and place of residence (during homecoming, etc.). Thus, the interview is conducted online or face-to-face. After the registration of participants had started, the progress was reported and the status was confirmed at a joint researcher’s meeting once a month.

### Conventional teaching materials

In the control group, the conventional teaching materials (alcohol handbook for university students) [33] will be handed over to the participants during the consent procedure and requested to be read. The survey will only be conducted for half a year by providing the conventional materials.

The main contents of the conventional materials are also included in the abovementioned test result report and health guidance materials for intervention. Although some descriptions are duplicated, the main contents are as follows:

- A recall of experiences such as the loss of a child or parent resulting from a fatal accident or illness caused by drinking alcohol, or a dangerous experience
- Current drinking style of university students (results of the drinking attitude survey)
- Conversion of the amount of alcohol consumed on the day of drinking according to a conversion table and checking of the current amount of alcohol consumed by using the alcohol consumption ranking table (male, female)
- Stage from tipsy to death resulting from acute alcohol intoxication
- Binge drinking
- Checking, defining, and coping with alcohol and harassment
- Specific examples of ideas for keeping tipsy at drinking parties
- Knowledge of alcohol (alcohol-related problems: violence, depression, sleep-related problems, family and workplace problems, mental and physical illnesses caused by alcohol, etc.)

## Ethics and dissemination

This research began in July 2021, with the approval of the Institutional Review Board of Medical Doctors, University of Tsukuba (Human Genome/Gene Analysis Research). If there are any changes in the research protocol, the principal researcher will apply to the Institutional Review Board of Medical Doctors, University of Tsukuba (Human Genome/Gene Analysis Research) for approval.

The researcher strictly manages the correspondence table that collates the participants with the ID number, and the data are managed so that the participant’s name and the information on the genotype constitution heard are not directly linked. Data input and analysis are mainly performed by the first author using a computer with security measures, and data collection and analysis results are regularly checked with collaborators.

This research was conducted in compliance with the “Ethical Guidelines for Human Genome/Gene Analysis Research” established by the Ministry of Health, Labor, and Welfare. Registration for the UMIN clinical trial was completed on June 1, 2021. The results of this research will be published in peer-reviewed academic journals and at internal and international academic conferences.

## Monitoring

Although this study is an intervention study, it is an educational intervention, and saliva testing is not considered invasive. Therefore, there is no data monitoring. To maintain compliance, we collaborate with researchers to attend monthly online meetings to share progress, data collection, and intermediate analysis results and discuss issues. Regarding the decision to suspend the recruitment of participants, the progress of the trial will be shared at the meeting, and if there is no problem in conducting the exam, the recruitment will be suspended when the number of subjects to be analyzed reaches the target. This research is subject to progress checks and audits every 6 months by the Institutional Review Board, and the person in charge of the audit is independent of this study.

## Risks, burdens, and benefits

Participation in this study is considered of little risk. However, 6 months of cooperation surveys, answering questionnaires, and receiving interventions are a small burden. As a benefit of participation, knowing the genetic predisposition of alcohol-metabolizing enzymes can lead to better management of drinking behaviors and reduced risks associated with drinking. On the contrary, information about the health risks associated with drinking may lead to participant anxiety. We will provide contact information for follow-up consultation in the case of mental burden. In the unlikely occurrence of an adverse event, participants will be instructed to contact the researcher, who will notify the responsible researcher’s doctor and take appropriate action, and will report to the department of the Institutional Review Board of Medical Doctors, University of Tsukuba (Human Genome/Gene Analysis Research) as necessary.

## Discussion

Information on the genetic function and constitution of alcohol-metabolizing enzymes provided individually by the study’s intervention may lead to a transition to a period of interest, such as a partial review of drinking methods, from indifference that participants perceive alcohol drinking as personal and objective information rather than general health information and are not aware of alcohol or hazardous drinking. At least in Japan, where the proportion of low *ALDH2* activity (a genetic component peculiar to East Asians) is high [1, 2], genetic polymorphism information of alcohol-metabolizing enzymes may be utilized for health management and risk prevention. Particularly, the prevention of lifestyle-related diseases for each *ALDH2* polymorphism has been elucidated as follows [34]: ALDH2 * 2 allelic carriers have a higher risk of carcinogenesis than non-ALDH2 * 2 but are less likely to increase blood pressure and have relatively lower serum liver transaminase (AST: aspartate transaminase, ALT: alanine transaminase, GGT: γ-glutamyl transpeptidase) and uric acid levels.

Moreover, ALDH2 * 2 allelic carriers may not have an opportunity to monitor their drinking habits [34]. Therefore, if a genetic predisposition delays the detection of abnormal biological indicators even with excessive drinking, detailed information on gene polymorphisms of alcohol-metabolizing enzymes in patients with lifestyle-related diseases may serve as an indicator to consider their drinking habits.

As previously mentioned, carriers of *alcohol dehydrogenase 1B* (ADH1B * 1 / * 1 genotype) have been reported to be at an increased risk of alcohol dependence [2], as well as an increased risk of head and neck cancer caused by alcohol consumption [6–8]. Previous studies conducted interventions involving only *ALDH2* genotype information [13, 19]. In this study, we propose a novel brief intervention that classifies the types of *ALDH2* and *ADH1B* gene polymorphisms into five combinations in an easily comprehensible manner. However, the effectiveness of health guidance using the information on *ALDH2* and *ADH1B* gene polymorphisms needs to be verified by increasing the sample size. Regarding the strength of this study, we will investigate the intervention effects of new efforts using the method of randomized controlled trials.

As mentioned in the introduction, a randomized controlled trial using the genetic information of the alcohol-metabolizing enzyme *ALDH2* validated the effect 30 days after providing Internet-based *ALDH2* genotype and health risk information [13]. In this previous study, the frequency and amount of drinking were significantly reduced after 30 days in the feedback group with low *ALDH2* activity. However, their follow-up period was 30 days, which is shorter than 6 months (maintenance period) [28], for behavioral habituation and maintenance. Therefore, the strength of the current study is the investigation of the intervention effect until 6 months (maintenance period) [28], which is the standard duration for behavioral habituation, and verification of its long-term efficacy.

Conversely, one limitation of this study is that when students agree to participate, they already know if they are in the group to be tested or not; hence, they cannot be blinded. Regarding the primary outcome measure, the average drinking amount on the drinking day is limited to the calculation of the amount of drink with an unknown alcohol concentration or amount. In addition, given that this research deals with genetic information, we need to evaluate the intervention effect by interviewing each participant. We also need to examine a system that can be useful for enrolling large groups, such as university students.

## Future contribution and significance of this research

This study will contribute to developing intervention methods, including the improvement of lifestyle habits using genetic polymorphic information for managing drinking problems.

## Trial status

The study was firstly registered in UMIN Clinical Trials Registry (https://www.umin.ac.jp/ctr/index.htm, R000050379) on June 1, 2021. The present protocol version 2 was changed to correct misdescriptions on April 16, 2022. The first participant was registered on July 20, 2021. Recruitment will be completed on March 31 2023.

## Data Availability

Any data required to support the protocol can be supplied on request with the approval of the participants. We are currently recruiting and collecting data. Datasets generated and/or analyzed during the current study are not published and are not available, but after the data collection is complete, it is available from the corresponding author and the first author upon reasonable request (in Japanese). The datasets and statistical codes analyzed during the current study are available from the corresponding authors upon reasonable request, as well as the full protocol.

## Abbreviations

AUDIT: Alcohol Use Disorder Identification Test
BASICS: Brief Alcohol Screening and Intervention for College Students
SBI: Screening and brief intervention
